# Abnormal Uroguanylin Immunoreactive Cells Density in the Duodenum of Patients with Diarrhea-Predominant Irritable Bowel Syndrome Changes following Fecal Microbiota Transplantation

**DOI:** 10.1155/2020/3520686

**Published:** 2020-02-04

**Authors:** Tarek Mazzawi, Øystein Eikrem, Gülen Arslan Lied, Trygve Hausken

**Affiliations:** ^1^Section of Gastroenterology, Department of Medicine, Haukeland University Hospital, Bergen, Norway; ^2^Department of Clinical Medicine, University of Bergen, Bergen, Norway

## Abstract

Altered densities of enteroendocrine cells play an important role in patients with irritable bowel syndrome (IBS). Uroguanylin activates guanylate cyclase-C to regulate intestinal electrolyte and water transport. *Aim*. To quantify uroguanylin immunoreactive cells density in the duodenum of diarrhea-predominant IBS (IBS-D) patients compared to controls and to investigate the effect of fecal microbiota transplantation (FMT) on these cell densities. *Method*. Twelve patients with IBS-D according to Rome III criteria were included. The cause was identified as post infectious (PI, *n* = 6) or idiopathic (*n* = 6). They completed the IBS-symptom questionnaire before and 3 weeks after FMT. Thirty grams of fresh feces donated from healthy relatives were diluted with 60 ml normal saline and instilled via endoscope into the duodenum. Biopsies were taken from the patients' duodenum before and 3 weeks after FMT. Duodenal biopsies taken from eight healthy controls were also included. The biopsies were immunostained for uroguanylin and quantified using computerized image analysis. *Results*. Uroguanylin immunoreactive cells were found both in duodenal villi and crypts in both controls and IBS-D patients. The densities of uroguanylin immunoreactive cells were significantly lower in the villi (*P* < 0.0001) and higher in the crypts (*P* < 0.0001) for the patients than the controls. Following FMT, the densities of uroguanylin immunoreactive cells for the total group and idiopathic subgroup decreased significantly in the duodenal crypts (*P* = 0.049 and 0.04, respectively) but not in the villi. No significant changes were shown in the PI-IBS subgroups. The cells density in only the crypts correlated with diarrhea (*r* = 0.97, *P* = 0.001) and bloating (*r* = –0.91, *P* = 0.01) in the PI-IBS subgroup before FMT and with abdominal pain (*r* = 0.63, *P* = 0.03) in the total group of IBS-D patients after FMT. *Conclusion*. Altered uroguanylin immunoreactive cells density was found in IBS-D patients compared to controls. Changes in these cells density following FMT correlated with IBS symptoms (diarrhea, bloating, and abdominal pain).

## 1. Introduction

Irritable bowel syndrome (IBS) is a chronic disorder of the gastrointestinal tract with an unclear cause and several contributing factors such as diet [1], infections [2, 3], genetics [4], gut microbiota [5, 6], and gut hormones [7, 8]. Interactions of the above-mentioned contributing factors with each other have certain effects on the gut-brain axis that may contribute to the development of symptoms related to the gastrointestinal [9, 10] and nervous [11, 12] systems. Previous publications have shown the effect of dietary changes on the symptoms [1, 13–16], gut microbiota [17, 18], and enteroendocrine cells [19–21] in IBS patients. In addition, changing the composition of the gut microbiota through fecal microbiota transplantation (FMT) had a positive clinical outcome not only in cases of *Clostridium difficile* [22, 23] but also in IBS [24–28].

**Figure 1:**
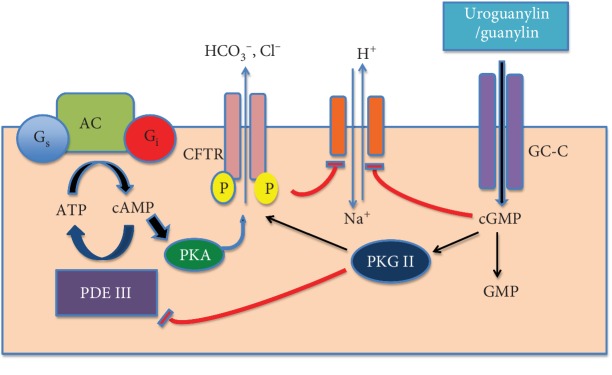
Activation cascade of binding of uroguanylin to guanylate cyclase-C receptor on the intestinal epithelial cell. Binding of uroguanylin to guanylate cyclase-C results in receptor activation, catalyzing the production of cyclic guanosine monophosphate (cGMP). Cyclic GMP can activate cGMP-dependent protein kinase II (PKGII) or inhibit the activity of a cyclic adenosine monophosphate- (cAMP-) specific phosphodiesterase, PDE III, thereby crossactivating cAMP-dependent protein kinase (PKA). PKGII and PKA phosphorylate the cystic fibrosis transmembrane conductance regulator or CFTR, increasing its chloride-secreting activity and preventing the absorption of sodium [37].

The gut neuroendocrine system regulates all the functions of the gastrointestinal tract and consists of enteroendocrine cells and the enteric nervous system [29]. Enteroendocrine cells are specialized cells that spread among the intestinal epithelial cells in both villi and crypts [29]. They have specialized microvilli that project into the gut lumen to act as sensors for the luminal contents and respond to luminal stimuli by releasing hormones that generally target other parts of the digestive system [29]. The guanylin peptide family includes guanylin, uroguanylin, lymphoguanylin, and renoguanylin and is proposed to function as intestinal natriuretic peptides [30]. Uroguanylin, encoded by the *GUCA2B* gene [31, 32], is a 16 amino acid peptide that is secreted by duodenal and proximal small intestinal enterocytes [33]. Uroguanylin acts as an agonist of the guanylyl cyclase receptor guanylate cyclase-C (GC-C) [34–36] by which its activation results in catalyzing the production of cyclic guanosine monophosphate (cGMP) (Figure 1) [37], hence regulating a variety of key processes such as chloride and bicarbonate secretion [37–39], epithelial cell growth, intestinal barrier integrity, and visceral sensitivity [39].

Newly published studies by our group [26, 28] showed that using FMT in patients with diarrhea-predominant IBS (IBS-D) improved their symptoms and changed their gut microbiota profile. Using the same study cohort, the aims of this study were to determine whether there is abnormality in the density of uroguanylin immunoreactive cells in the duodenum of IBS patients compared to healthy controls (controls), to study the effect of changing the gut microbiota through FMT on the density of these cells, and to find the correlations between these cells and IBS symptoms (if any).

## 2. Methods

### 2.1. Patients, Donors, and Controls

Patients (*n* = 16) who were referred to the outpatient clinic of gastroenterology, Haukeland University Hospital, Bergen, Norway, met the Rome III criteria for IBS, and scored >175 for the IBS-Symptom Severity Scoring system (IBS-SSS) were included in the current study.

Patients who had a history of inflammatory bowel diseases, GI malignancy, blood in stool, oral thrush, disseminated lymphadenopathy, underwent abdominal surgery, and pregnant or lactating women were excluded. In addition, the exclusion criteria included immunocompromised patients or those taking immunosuppressive medications, had history of opportunistic infections within 1 year, or took probiotics or antibiotics within 1 month prior to fecal transplantation.

Feces donors were healthy family members not complaining of IBS and were 7 males and 9 females with an age range of 20-55 (mean age 35) years. The exclusion criteria for the donors were the same as for the patients.

Biopsy samples from a group of 12 healthy volunteers, 10 females and 2 males with an age range of 20-42 (mean age 39) years, recruited at Stord Hospital, Stord, Norway, by advertising in the local newspapers, were used as controls to study duodenal uroguanylin immunoreactive cells.

The study was performed in accordance with the Declaration of Helsinki [40] and was approved by the Regional Committee for Medical and Health Research Ethics in Western Norway (reference no.: 2013/1497). The current study was registered at ClinicalTrials.gov (ID: NCT03333291). All participants provided written informed consent. All the data concerning uroguanylin immunoreactive cell density for the current study are presented in this article.

### 2.2. Study Design

As previously explained in details [26], the donors and patients underwent screening one week before FMT and were physically examined and blood tested to exclude contagious infectious agents for the donors (hepatitis A, B, and C; HIV; Epstein-Barr virus; and cytomegalovirus) and organic disorders for the patients according to previously published recommendations [22, 41, 42]. Stool samples from both donors and patients were examined for fecal calprotectin and screened for bacteria, viruses, parasites, and eggs. The patients were instructed not to change their lifestyle or diet and to report of any change in their life style or use of new medications before the start or during the study.

### 2.3. FMT Procedure

Thirty grams of fresh feces collected from the donors (<2 hours from production to donation) were manually mixed with 60 ml normal saline and sieved for preparing fecal suspension without any hard particles [22, 41, 42], on the transplantation day. After an overnight fast, 60 ml of fecal suspension followed by 60 ml normal saline were flushed only once through the endoscope into the descending part of the patients' duodenum. Tissue biopsies were taken from the descending part of the duodenum before installation of feces and again after 3 weeks. Fecal samples were collected on the same day of FMT (before the procedure) and 3 weeks after FMT and sent for microbiota analysis using the GA-map™ Dysbiosis Test (Genetic Analysis AS, Oslo, Norway) as previously explained in details [26].

### 2.4. Questionnaires

The following questionnaires for IBS symptom assessment were completed by the patients before and 3 weeks after FMT: (a) IBS-Symptom Severity Scoring system (IBS-SSS scores < 175 represent mild IBS symptoms, 175–300 represent moderate severity, > 300 represent severe IBS) by which a decrease of 50 points correlates with improvement in clinical symptoms [43] and (b) IBS-symptom questionnaire (IBS-SQ) which reports each of IBS symptoms (nausea, bloating, abdominal pain, constipation, diarrhea, and anorexia) using a severity scale from 0 to 10, where 0 = no symptoms and 10 = severe symptoms [44, 45].

### 2.5. Gastroscopy and Immunohistochemistry

The patients fasted overnight prior to undergoing a gastroscopy. During the gastroscopy, four tissue biopsies were collected from the descending part of the duodenum, distal to the papilla. The biopsy samples were fixed in formaldehyde and embedded in paraffin. The biopsies were cut into 3 *μ*m thick sections. Antigen retrieval was performed in the PT-Link® system for 20 minutes at 98°C with the Dako® EnVision FLEX Target retrieval buffer at pH 6 (K8005; Dako, Jena, Germany). Peroxidase blocking solution was used for 10 minutes. Thereafter, further blocking with 5% goat serum (X0907; Dako) in 3% bovine serum albumin for 30 minutes was performed. A one-hour incubation with a 1 : 50 dilution of the primary polyclonal rabbit antibody to guanylate cyclase type B was used (GUCA2B, no. LS-C371347, LifeSpan BioSciences Inc., Seattle, WA, USA). The secondary EnVision HRP anti-rabbit (K4011; Dako) antibody was incubated for 30 minutes. The slides were incubated with 3,3′-diaminobenzidine (DAB) solution for 8 minutes followed by counterstaining with hematoxylin.

### 2.6. Computerized Image Analysis

Uroguanylin immunoreactive cells density was quantified using a light microscope with ×40 objective and a computer software Cell^B imaging program (Olympus, Tokyo, Japan). By keeping the identity of the slides concealed, the quantification was performed by T.M. in 10 nonoverlapping fields. Each field (frame) of epithelial cells represents a tissue area measured at 0.09 mm^2^. The density of uroguanylin immunoreactive cells in the duodenal villi was expressed as the number of cells/100 epithelial cells and in duodenal crypts as the number of cells/mm^2^ of epithelium.

### 2.7. Gut Microbiota Analysis

The analysis was previously described in details [26]. Briefly, GA-map™ Dysbiosis Test is based on fecal homogenization and automated total bacterial genomic DNA extraction using magnetic beads. Fifty-four DNA probes were used to target bacterial strains based on their 16S rRNA sequence in seven variable regions (V3–V9). Twenty-six probes detect specific species, 19 probes detect bacteria on the genus level, and 9 probes detect bacteria at higher taxonomic levels. The signal detection was performed by using a BioCode 1000A 128-Plex Analyzer (Applied BioCode, Santa Fe Springs, CA, USA) [46].

## 3. Statistical Analysis

Statistical analysis was performed using the GraphPad Prism 7 (GraphPad Software, Inc.). Normal distribution of data was tested using the D'Agostino-Pearson omnibus normality test. Kruskal-Wallis with Dunn's post hoc test was used to compare uroguanylin immunoreactive cells density between the controls and the patients before and after receiving FMT. Paired *t-*test is used to compare between the IBS-SQ symptoms and cell densities of the patients before and after receiving FMT. Mann-Whitney *U* test was used to compare the gut microbiota profiles of the patients before and after FMT to their respective donors. Correlations were performed using Pearson's test for parametric and Spearman's test for nonparametric datasets. The data are presented as mean ± SEM values. *P* < 0.05 are considered to be statistically significant.

## 4. Results

Twelve patients (4 females and 8 males, with age range of 20-44 years) completed the study after excluding four patients due to withdrawal of the participation consent for practical reasons (*n* = 1), failed intubation of the endoscope during the gastroscopy session 3 weeks after FMT (*n* = 1), positive stool culture for *Clostridium difficile* (*n* = 1), and being diagnosed with functional dyspepsia (*n* = 1). Six patients suffered from post infectious (PI) IBS and six had idiopathic IBS. They completed the whole study by filling out the questionnaires and delivered stools for microbiota analysis on the same day of undergoing gastroscopy with duodenal biopsies, i.e., the day of FMT, and after 3 weeks. No change in lifestyle, diet, or use of any/new medications has been registered during the study.

### 4.1. Symptom Questionnaires

The total scores for IBS-SSS and IBS-SQ significantly improved as previously reported [26]. The scores for IBS-SQ showed significant improvements in several of its domains in both groups of IBS as shown in Table 1.

### 4.2. Gastroscopy, Histopathology, Immunohistochemistry, and Image Analysis

The duodenum of the patients was both macroscopically and microscopically normal.

**Figure 2:**
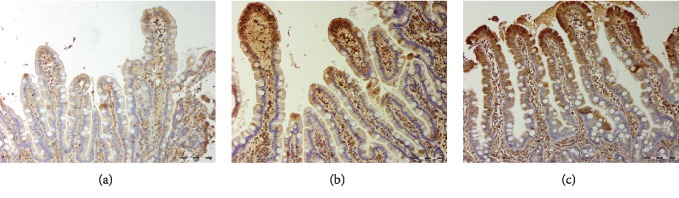
Uroguanylin immunoreactive cells in the duodenal villi for patients with irritable bowel syndrome before (a) and after (b) fecal microbiota transplantation and controls (c).

**Figure 3:**
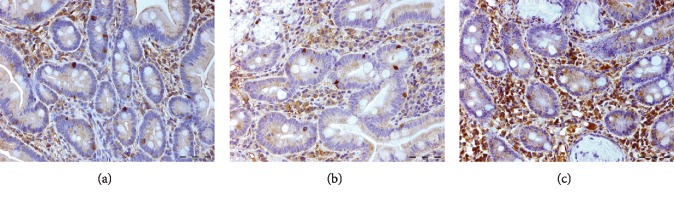
Uroguanylin immunoreactive cells in the duodenal crypts for patients with irritable bowel syndrome before (a) and after (b) fecal microbiota transplantation and controls (c).

Uroguanylin immunoreactive cells were found in both the villi (Figure 2) and crypts (Figure 3) of the duodenum, which is consistent with a previous publication [47], in both controls and IBS patients. The staining in the villi was concentrated mainly in the cytoplasm of the epithelial cells. Uroguanylin immunoreactive cells in the crypts, identified as enteroendocrine cells, were either basket- or flask-shaped [48].

**Figure 4:**
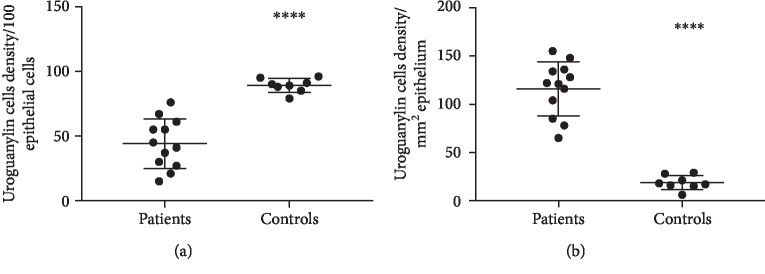
Uroguanylin immunoreactive cells densities in the duodenal (a) villi and (b) crypts, for patients with irritable bowel syndrome and controls.

At baseline, the densities of uroguanylin immunoreactive cells were significantly lower in the villi (*P* < 0.0001) and higher in the crypts (*P* < 0.0001) for the patients than the controls (Figures 4(a) and 4(b), respectively). Three weeks after FMT, the densities of uroguanylin immunoreactive cells for the total group and idiopathic IBS subgroup decreased significantly in the duodenal crypts (*P* = 0.049 and 0.04, respectively) but not in the villi (*P* = 0.5 and 0.8, respectively, Table 2). No significant changes were shown in either duodenal crypt or villi in the PI-IBS subgroups, Table 2.

### 4.3. Gut Microbiota

The total changes occurring to the gut microbiota profiles for the donors and the total group of IBS patients before and after FMT were previously described [26, 28]. Briefly, several strains of the gut microbiota, namely, *Ruminococcus gnavus*, *Clostridium sensu stricto*, *Actinobacteria*, *Bifidobacteria*, and *Gardnerella*, for the donors were statistically significantly different from those for the patients before FMT, which normalized for the patients 3 weeks after FMT. No statistically significant differences were found in the bacterial strains when comparing the patients before vs. after FMT.

### 4.4. Correlations

**Figure 5:**
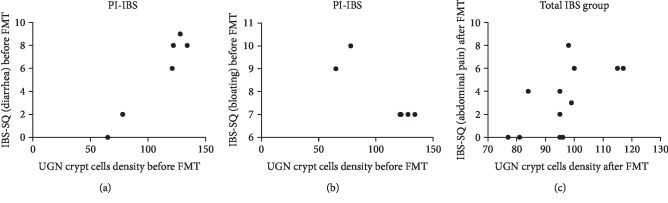
Correlations between scores of irritable bowel syndrome-symptom questionnaire (IBS-SQ) for (a) diarrhea, (b) bloating, and (c) abdominal pain, and uroguanylin (UGN) immunoreactive cells density of the crypt in post infectious (PI-IBS) and total group of patients with irritable bowel syndrome before and after fecal microbiota transplantation (FMT).

Using Pearson's test, uroguanylin immunoreactive cells density before FMT correlated positively with diarrhea (*r* = 0.97, *P* = 0.001) and negatively with bloating (*r* = −0.91, *P* = 0.01) in the PI-IBS subgroup (Figures 5(a) and 5(b), respectively). After FMT, uroguanylin cells densitycorrelated positively with abdominal pain (*r* = 0.63, *P* = 0.03) in the total group of IBS patients (Figure 5(c)). No correlations were found between the cells in the villi and IBS symptoms.

Using Spearman's correlation test, significant correlations were found between several bacterial strains and uroguanylin immunoreactive cells density in the duodenal villi and crypts of patients in the total group of IBS patients before and after FMT as shown in Table 3. These cells density also correlated with *Bacteroides fragilis* and *Escherichia*/*Shigella* in the duodenal villi and crypts, respectively, before FMT in the PI-IBS group. No correlations were found between the uroguanylin immunoreactive cells density and gut microbiota in the idiopathic IBS group. The gut microbiota profiles that correlated with the uroguanylin immunoreactive cells density are shown in Table 3.

## 5. Discussion

The current study describes the differences in the uroguanylin immunoreactive cells density between IBS-D patients and controls. We also investigate how the cells density changes following FMT and correlates to IBS symptoms and gut microbiota before and after FMT.

Gut hormones, which control several functions of the gastrointestinal tract [49, 50], are released by the enteroendocrine cells of the gut for which alterations in the densities of these cells have also been postulated to play an important role in the pathophysiology of IBS [29, 51–55]. To our knowledge, the differences in the uroguanylin immunoreactive cells density between patients with IBS-D and controls have never been described before. Significant differences in the densities of uroguanylin immunoreactive cells were found between IBS-D patients and controls at baseline, and only the density of the cells in the crypts significantly changed following FMT. It is difficult to explain why the changes in the density of uroguanylin immunoreactive cells were specific only to those in the crypts following FMT; however, one may speculate that the cells in the villi may react to different stimuli than those they were exposed to in the current study.

Uroguanylin belongs to the guanylin peptide family that serves as a paracrine/endocrine hormone and acts as agonists of the guanylyl cyclase receptor GC-C, which regulates intestinal electrolyte and water transport [30, 35]. The activation of these receptors by the small heat-stable enterotoxins produced by the enterotoxigenic *Escherichia coli* causes an increase in chloride ion secretion, leading to secretory diarrhea [56, 57]. This explains the correlations between uroguanylin immunoreactive cells density and diarrhea in PI-IBS patients at baseline before FMT. Correlations between uroguanylin and other IBS symptoms such as bloating and abdominal pain are most probably due to the same mechanism of the GC-C and cGMP pathway activation leading to reduced visceral hypersensitivity [58, 59], thus linking it to the gut-brain axis. Plecanatide®, a medication structurally related to uroguanylin, has newly been introduced to the market for the treatment of abdominal pain and constipation in patients with constipation-predominant IBS [60].

Currently, no data is present to explain the correlations between uroguanylin cells and other bacterial strains other than those that produce small heat-stable-like toxins (other *Escherichia coli* species, *Yersinia enterocolitica*, *Vibrio cholerae*, *Salmonella* species, and *Klebsiella pneumoniae*) [61]. However, *Bacteroides fragilis*, which correlates with uroguanylin cells in the crypts, is a bacterium that disrupts tight junctions by proteolytic degradation of tight junction proteins using enterotoxin or fragilysin leading to diarrhea [61]. This toxin is more potent when it acts on the basolateral rather than the apical part of the epithelial surface [62].

The relationship between uroguanylin and guanylin and their receptor is not limited to gastrointestinal disorders but also involves renal disorders, colorectal cancer, metabolic syndrome, and mental disorders among others [39]. Recent studies identified a novel role for uroguanylin in obesity by regulating satiety via the gut-brain axis [47].

The current study has several limitations such as the small number of the participants and the lack of a placebo-controlled group from which tissue biopsies could have been taken from. For this exploratory and explanatory study, we aimed to study if there were any differences in the uroguanylin enteroendocrine cells between the different subgroups of IBS-D patients. Despite the small sample size of the different IBS-D subgroups, we found significant differences in uroguanylin immunoreactive cells density before and after FMT; however, further studies with larger groups of participants are required to be able to draw firm conclusions.

## 6. Conclusions

Altered uroguanylin immunoreactive cells density has been found in IBS-D patients compared to controls. Changes in these cells density following FMT correlated with IBS symptoms (diarrhea, bloating, and abdominal pain). The current study shows that both gut microbiota and the enteroendocrine cells play an important role in the pathophysiology of IBS.

## Figures and Tables

**Table 1:** Scores of the irritable bowel syndrome-symptom questionnaire in post infectious and idiopathic irritable bowel syndrome before and after fecal microbiota transplantation.

IBS-SQ questionnaire	Post infectious IBS		*P* value^∗^	Idiopathic IBS		*P* value^∗∗^
	Before FMT	3 weeks after FMT		Before FMT	3 weeks after FMT	
Nausea	2 ± 0.7	1 ± 0.6	0.2	5.3 ± 1.4	1.8 ± 0.98	**0.002**
Bloating	7.8 ± 0.5	3 ± 1.2	**0.004**	8 ± 1	3.8 ± 1.3	**0.003**
Abdominal pain	6.3 ± 1.4	4.3 ± 1.2	0.13	6.2 ± 1.2	2.2 ± 1	**0.003**
Constipation	2.7 ± 0.95	1.2 ± 0.8	0.3	5.8 ± 1.9	1.8 ± 1.2	0.1
Diarrhea	5.5 ± 1.5	1.2 ± 0.6	**0.01**	7.3 ± 0.95	1.2 ± 0.5	**0.001**
Anorexia	1 ± 0.5	1 ± 0.5	>0.99	3.5 ± 1.2	0.8 ± 0.5	**0.038**

Comparison: Paired *t*-test. Data are presented as the mean ± SEM. ^∗^Post infectious IBS before vs. after FMT, ^∗∗^idiopathic IBS before vs. after FMT. FMT: fecal microbiota transplantation; IBS-SQ: irritable bowel syndrome-symptom questionnaire.

**Table 2:** Densities of uroguanylin immunoreactive cells in the duodenum of patients with irritable bowel syndrome before and after fecal microbiota transplantation.

	Uroguanylin immunoreactive cells density		
	Before FMT	After FMT	*P* value
*Total IBS group*			
Villi (cells/100 epithelial cells)	44 ± 5.5	41 ± 2.3	0.5
Crypts (cells/mm^2^)	116 ± 8	96 ± 3	**0.049**
*PI-IBS*			
Villi (cells/100 epithelial cells)	45.8 ± 7.4	40.5 ± 3.3	0.6
Crypts (cells/mm^2^)	108 ± 11.8	102.5 ± 4	0.6
*Idiopathic IBS*			
Villi (cells/100 epithelial cells)	42.5 ± 8.8	40.7 ± 3.6	0.8
Crypts (cells/mm^2^)	124 ± 11	89.5 ± 4	**0.04**

Comparison: Paired *t*-test. Data are presented as the mean ± SEM. IBS: irritable bowel syndrome; PI: post infectious.

**Table 3:** Gut microbiota profiles that correlated with uroguanylin immunoreactive cells density in the duodenal villi and crypts of patients with irritable bowel syndrome before and after fecal microbiota transplantation.

Bacterial strain	Bacterial signals		*P* ^∗^	Correlations with uroguanylin cells density (*r*, *P*^∗∗^)	
	Before FMT	After FMT		Before FMT	After FMT
Total IBS group					
*Bacteroides fragilis*	39 ± 23	37 ± 16	0.72	Crypts (-0.81, 0.02)	—
*Parabacteroides*	7.9 ± 3	14.6 ± 5	0.24	—	Crypts (0.796, 0.01)
*Alistipes*	107 ± 25	127 ± 27	0.2	—	Crypts (0.71, 0.038)
*Streptococcus sanguinis* and *S. thermophilus*	171 ± 80	78 ± 42	0.59	—	Villi (0.69, 0.047)
PI-IBS group					
*Bacteroides fragilis*	65 ± 26	36 ± 28	0.4	Villi (0.94, 0.02)	—
*Escherichia/Shigella*	141 ± 104	181 ± 135	0.4	Crypts (0.94, 0.02)	—

Comparison: Paired *t*-test. Data are presented as the mean ± SEM. Spearman's correlation test (*r*) was used to correlate between the bacterial strain and uroguanylin immunoreactive cells density in the duodenal villi and crypts. ^∗^Comparison between the signals before and after FMT; ^∗∗^*P* values for correlations. FMT: fecal microbiota transplantation; IBS: irritable bowel syndrome; PI: post infectious.

## Data Availability

“All the data concerning uroguanylin immunoreactive cells density for the current study are presented in this article.” So the data used to support the findings of this study are included within the article. A study protocol is already published as a supplementary in PLOS One with the provided doi: 10.1371/journal.pone.0194904.s00.
